# Pre-endoscopy drink of small doses of simethicone and pronase solution improves visualization in upper gastrointestinal endoscopy: Randomised double-blind experiment

**DOI:** 10.1097/MD.0000000000046271

**Published:** 2025-11-28

**Authors:** Yuan Li, Jingping Jiang, Jinyi Sheng, Junmin Wu

**Affiliations:** a Division of Gastroenterohepatology, Department of Internal Medicine, The First People’s Hospital of Jingde Zhen, Jingdezhen, Jiangxi, China.

**Keywords:** dyclonine, endoscopy, premedication, pronase, simethicone

## Abstract

**Background:**

Upper gastrointestinal endoscopy (UGE) was the gold standard investigative modality for gastrointestinal diseases. High quality of visualization was essential to high-quality UGE. Our aim was to compare the effect of the small doses of simethicone plus pronase in improving visibility during UGE.

**Methods:**

Four hundred patients underwent UGE and were divided into 4 groups randomly. All patients took medicine 15 to 30 minutes before the examination. The premedication in the group D was dyclonine. The other 3 groups were administered simethicone (group S), pronase solution (group P) alone, and simethicone plus pronase solution (group P + S). The mucosal visibility score and total mucosal visibility score (TMVS) were recorded. The lower the score, the cleaner the gastric mucosa. Adverse events were also recorded.

**Results:**

We studied the UGE pictures of 400 patients. The group P + S had a significantly better TMVS than the other groups (4.91 ± 0.99 in group P + S vs 6.76 ± 1.58 in group D, *P* < .05; 4.91 ± 0.99 in group P + S vs 5.61 ± 0.91 in group S, *P* < .05; 4.91 ± 0.99 in group P + S vs 6.46 ± 0.82 in group P, *P* < 0. 05). However, there was no significant differences were found between group D versus group P on TMVS.

**Conclusion:**

The application of small doses of simethicone and pronase solution can create good mucosal and has no side effects.

## 1. Introduction

Gastric cancer is the second most common malignancy in China and is the fourth leading cause of cancer-related death in the world.^[1]^ Many patients are diagnosed in the advanced stage which will bring great shock to patients financially and mentally. Advanced gastric cancer has a poor prognosis with 5-year survival for stage 4 disease of 5.2%.^[2]^ Early diagnosis and treatment greatly improve the survival rate and quality of life in patients with gastric cancer.^[3]^

Therefore, it is imperative that it be identified at as early a stage as possible. High quality in upper gastrointestinal endoscopy (UGE) is a most important step in finding the diagnostic yield in the detection of premalignant and early malignant lesions. We always use narrow-band imaging, blue laser imaging, or linked color imaging to detection and further examination of gastritis and early gastric cancer. However, clustered mucus, foam, and bubbles in the UGE severely affect mucosal visibility,^[4–12]^ leading to missed lesions and misdiagnosis. To get better visualization, prescribing premedication to get rid of clustered mucus, foam, and bubbles is necessary. Nevertheless, the choice of premedication in different endoscopic centers in China is not completely consistent. The main aim of the study is to assess which group has better visualization.

## 2. Materials and methods

1. We enrolled a consecutive series of patients ranging in age from 18 to 70 years who were referred to the Digestive Endoscopic Center of the First People’s Hospital of Jingde Zhen, Jingdezhen, Jiangxi for UGE. Patients with any of the following conditions were excluded: a contraindication for UGEs; pregnancy or breastfeeding; an allergy to dyclonine, pronase, or simethicone; severe heart or respiratory condition, liver dysfunction, or other life-threatening diseases; liver cirrhosis with esophageal and gastric varices; active gastrointestinal bleeding; a history of gastrointestinal surgery; severe gastric retention; refusal to participate in this study; and current participation in other clinical trials and in the follow-up or drug washout period.
2. Patients were divided into 4 treatment groups: Group D (dyclonine 10 mL + water 70 mL); Group S (simethicone 10 mL + water 70 mL) (200 mg/5-mL solution); Group P (Pronase + water: 80 mL of water and 20,000 IU pronase granules combined with 1-g NaHCO3) (1250 U/5-mL solution); and Group S + P (pronase + simethicone water) (pronase: 1250 U/5-mL solution) (simethicone: 200 mg/5-mL solution), patients received 80 mL of water, 10 mL of simethicone, and 20,000 IU pronase granules combined with 1-g NaHCO3. All patients received standard recommendation instructions, which included 8 hours fast, and ingestion of the study solution under supervision 15 to 30 minutes before the procedure.
3. The following medicines were used in this study: dyclonine produced by Yangtze River Pharmaceutical Group Co., Ltd, pronase produced by Beijing Tide-Pharmaceutical Co., Ltd., Beijing, China, and simethicone produced by Guangcheng Pharmaceutical Group Co., Ltd. The following instruments were used in this study: a magnifying endoscope capable of magnification ×145 (e.g., −760Z; FUJIFILM Co. Ltd, Tokyo, Japan) and a standard videoendoscopic system (EVIS LUCERA; Olympus).

### 2.1. Endoscopic procedure and outcomes

**Figure 1.**
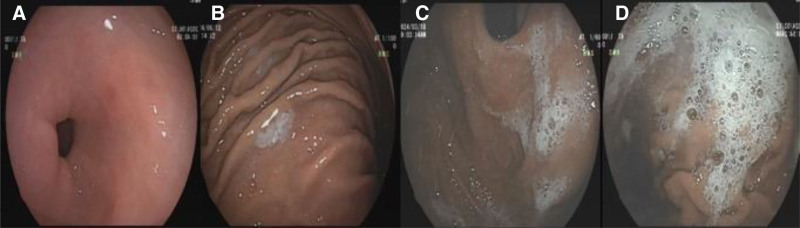
Endoscopic scoring system. (A) Score of 1: No bubbles. (B) Score of 2: Minimal bubbles which the endoscopist must actively look for. (C) Score of 3: Foam is obviously present but not severe. (D) Score of 4: Severe foam obscuring vision.

After intubation of stomach and duodenum, electronic photographs were captured as per standard reporting followed by our department at 4 predefined locations gastric fundus, gastric body, gastric antrum, and duodenal bulb without any interventions. The primary outcome was the overall gastric mucosa visibility, using the total mucosal visibility score (TMVS). Each area had a score:1 (No adherent mucus and clear views of the mucosa), 2 (A thin coating of mucus that did not obscure views of the mucosa), 3 (Some mucus/bubbles partially obscuring views of the mucosa, a small mucosal lesion might be missed without flushing), and 4 (Heavy mucus/bubbles obscuring views of the mucosa, a small mucosal lesion could easily be missed without flushing) (Fig. 1). The TMVS is the sum of the 4 scores, ranging from 4 to 16 points. Secondary outcomes included endoscopic and histological findings. All endoscopic and histological findings were recorded and subsequently categorized as major and minor findings. Biopsies were taken according to the endoscopist’s discretion. The following were considered to be major endoscopic findings: atrophy, metaplasia, granular gastropathy, ulcers, neoplasia, polyps, and erosions.

### 2.2. Statistical analysis

Statistical analysis was carried out using SPSS 22.0. Demographic characteristics were assessed using the χ2 test or Fisher exact test. The Shapiro–Wilk test was used to test the normality of quantitative data. The TVS and mucosal visibility score (MVS) are shown as the mean ± standard deviation. The Kruskal–Wallis test and Dunn multiple comparison correction were used to compare different premedication groups before esophagogastroduodenoscopy. *P *< .05 was considered statistically significant.

## 3. Results

### 3.1. Demographic characteristics of the patients

We enrolled 400 patients in the study, including 100 in each group. There were no statistically significant differences on the demographic (age and gender) (all *P*-values > .05) (Table 1). There were no complications or serious adverse events in any of the groups during the examinations.

**Table 1:** Baseline characteristics.

	Group D	Group S	Group P	Group P + S	*P*
Age	51.60 ± 13.91	49.43 ± 12.17	50.33 ± 12.37	51.01 ± 13.57	<.05
Male	49	51	50	49	<.05

### 3.2. Mucosal visibility scores

When evaluated for the primary objective of MVS, there were significant difference among the groups. The mean TMVS were significantly better in the group S + P (4.91 ± 0.99) compared with another 3 group S (5.61 ± 0.91), group D (6.76 ± 1.58), and group P (6.46 ± 0.82) (*P* < .05). However, there was no statistically significant differences between group D and group P (*P* = .80, *P* > .05). Meanwhile, the mean MVS was significantly better in group S + P than another 3 groups in the gastric fundus, gastric body, and gastric antrum. However, there is no statistically significant about MVS in the gastric body and gastric antrum between group D and group P (*P* > .05) (Table 2). In addition, there are only statistically differences between group D (1.11 ± 0.31) versus group S + P (1.00 ± 0.00) (*P* < .05) and group P (1.07 ± 0.26) versus group S + P (1.00 ± 0.00) (*P* < .05) in duodenum.

**Table 2:** Mucosal visibility scores by location.

Study group					*P*-value					
	Group D	Group S	Group P	Group S + P	D vs S	D vs P	D vs S + P	S vs P	S vs S + P	P vs S + P
Gastric fundus	2.11 ± 0.90	1.43 ± 0.72	1.62 ± 0.66	1.18 ± 0.38	*P* < .05	*P* < .05	*P* < .05	*P* < .05	*P* < .05	*P* < .05
Gastric body	2.27 ± 0.94	1.97 ± 0.97	2.45 ± 0.89	1.68 ± 0.78	*P* < .05	*P* > .05	*P* < .05	*P* < .05	*P* < .05	*P* < .05
Gastric antrum	1.27 ± 0.47	1.14 ± 0.35	1.32 ± 0.51	1.03 ± 0.17	*P* < .05	*P* < .05	*P* < .05	*P* < .05	*P* < .05	*P* < .05

Group D: Dyclonine solution.

Group S: Simethicone solution.

Group P: Pronase solution.

Group S + P: Simethicone plus pronase solution.

#### 3.2.1. Adverse events

No adverse events attributable to the pre-endoscopy drink were seen. All patients tolerated the procedure well and there were no instances of vomiting, aspiration, or intolerance.

## 4. Discussion

UGE was the gold standard for diagnosing gastric cancer. It was well documented that impaired mucosal visibility secondary to adherent mucus was a potential cause for incomplete UGE and thus missed early gastric cancer. Clear mucosal visibility can reduce the need for additional manipulation, such as extra washings, and can shorten total procedure time.

Our study demonstrated improved mucosal visualization on UGE after small doses of simethicone and pronase. The effect was highly significant as shown by statistical analysis. Simethicone was a silicone-based nonabsorbable material that causes gas bubbles to burst by reducing their surface tension.^[13,14]^ Pronase was a proteolytic enzyme with a pH range of 7.0 to 10.0 that had the effect of reducing the viscosity of gelatin and mucin, and the effect of reducing mucin in gastric mucus was strongest by cutting the peptide bonds of mucin, the main component of gastric mucus, dissolving, and removing gastric mucus. Therefore, from both theoretical and practical perspectives, the administration of simethicone combined with pronase before examination can improve the visualization of the gastric mucosa.

In our study, the concentration of pronase is 1250 U/5-mL solution, and the concentration of Simethicone is 200 mg/5-mL solution. The concentrations of premedication agents for gastrointestinal endoscopy were selected based on existing literature and clinical practicality. For pronase, a concentration of 1250 U/5 mL was adopted, as this dosage has been demonstrated to be effective and is consistently employed in the majority of relevant studies, including those by Liu X et al and Li Cao et al.^[15,16]^ Regarding simethicone, a concentration of 40 mg/mL was chosen for this study. While previous research, such as that by Shrihari Anil Anikhindi et al has shown that 40 mg/mL provides excellent mucosal visibility,^[17]^ and Esteban Fuentes-Valenzuela et al reported satisfactory results with a lower concentration of 2 mg/mL.^[18]^ Our decision was based on 2 primary considerations. First, our review of the literature indicated that the 40-mg/mL concentration is frequently utilized and is perceived to offer a more robust effect for ensuring optimal mucosal visualization compared with the lower dosage. Second, the standard commercial preparation available in our institution is 40 mg/mL per vial. Using this concentration directly streamlines clinical workflows, simplifies preparation, and enhances protocol adherence. It is acknowledged that while theoretically increasing the simethicone concentration might potentially improve efficacy, it could also lead to an unnecessary increase in healthcare costs without proven significant benefit. Therefore, the use of the standard 40-mg/mL concentration represents a balance between anticipated effectiveness and economic practicality.

While significant improvement in mucosal visibility was seen in all regions of stomach, insignificant difference was seen in duodenum in most part of groups. This was similarly reported by the studies of Elvas L et al^[7]^ and Ahsan et al.^[19]^ The reason was that there was no enough time for the mucus in the stomach to be discharged into the duodenum. Besides, we found that mucosal cleanliness was reduced only when there was lesion in the duodenum, such as duodenal ulcer, duodenocholangitis, et al.

Although P + S group was the group with the best gastric mucosal cleanliness, some patients have poor gastric mucosal cleanliness scores, which was due to the fact that the interval between taking medicine and starting the examination was too short.

The concentration of simethicone is anticipated to exert a significant influence on the experimental outcomes. It is important to note, however, that a higher concentration does not invariably yield superior efficacy, as the duration of its presence within the gastric cavity is also a critical determinant. Furthermore, even at an identical concentration, variations in the contact time will lead to differential effects.^[20]^ Mingjun Song et al^[21]^ found that it was best way to start gastroscopy at least 30 minutes after taking the drugs. However, a precise and specific time frame has not been established. Nevertheless, it is recommended that allowing sufficient reaction time for the medication can enhance gastric mucosal visibility. The exact duration required warrants further investigation. In addition, whether *Helicobacter pylori* infection affected the cleanliness of the gastric mucosa when using the same premedication before gastroscopy was also something we need to further explore.

In conclusion, we would recommend a pre-endoscopy drink of pronase with small doses simethicone prior to all elective UGE procedures. Our study suggests that usefulness of pronase with small doses simethicone can achieve satisfactory endoscopic viewing.

## Author contributions

**Data curation:** Yuan Li, Jingping Jiang, Jinyi Sheng, Junmin Wu.

**Formal analysis:** Yuan Li.

**Writing – original draft:** Yuan Li.

**Writing – review & editing:** Yuan Li.
